# Vacuoles in the Breast: A Histologic Clue for an Unusual Presentation of an Atypical Organism

**DOI:** 10.7759/cureus.36586

**Published:** 2023-03-23

**Authors:** Caitlin M Raymond, Marisa C Nielsen, Colleen Silva, Melinda Tanabe, Cecilia Clement, Natalie Williams-Bouyer, Jing He

**Affiliations:** 1 Pathology, University of Texas Medical Branch at Galveston, Galveston, USA; 2 Pathology and Laboratory Medicine, Boston Medical Center, Boston, USA; 3 Surgery, University of Texas Medical Branch at Galveston, Galveston, USA; 4 Infectious Disease, University of Texas Medical Branch at Galveston, Galveston, USA

**Keywords:** clinical microbiology, breast pathology, histopathologic examination, histopathology (hp), emerging pathogen, interdisciplinary health team, breast abscess, non-tuberculosis mycobacteria

## Abstract

Infections with nontuberculous mycobacteria (NTM) are increasing in prevalence worldwide, and this group of organisms is emerging as significant clinical pathogens. We present a case of a 58-year-old female with persistent furuncles of the breast who was found to have an NTM infection. This case is unique for the lack of risk factors for NTM in the patient’s history, the location of the infection in the breast, and the close cooperation needed across disciplines to arrive at the diagnosis. This multi-disciplinary discussion considers the classic clinical presentation of NTM, it is a characteristic morphological appearance on histopathology, the differential diagnosis, treatment, and the ultimate outcome of the case. This case report and discussion will assist both clinicians and pathologists in the diagnosis of this important infectious disease.

## Introduction

The genus *Mycobacterium *has over 190 recognized species [1], which include well-known human pathogens such as *Mycobacterium tuberculosis*, the causative pathogen of tuberculosis, and *Mycobacterium leprae*, the causative agent of leprosy. Over the past two decades, the number of known mycobacterial species has increased, with the majority of discoveries being classified as nontuberculous mycobacteria (NTM) [2]. Our understanding of NTM as human pathogens has greatly expanded: currently, NTM are known to cause a wide range of clinical diseases [3,4], including pulmonary disease, central nervous system disease, bacteremia, and disseminated disease in immunocompromised patients, as well as ocular, dermatological, and soft tissue infections [5]. One of the most pathogenic species of NTM responsible for human infection is *Mycobacterium abscessus* complex, so named for its association with subacute cutaneous infections with fistula formations and subcutaneous abscesses. This complex includes three subspecies: *M. abscessus *subsp*. abscessus*; *M. abscessus *subsp*. bolletii* and *M. abscessus* subsp. *massiliense*. Being ubiquitous in the environment, they are often found in soil, water, and dust [3]. Currently, the acquisition of *M. abscessus* infection is not well understood, involving not only host risk factors, but also environmental exposures and subspecies-dependent virulence [6,7].

Originally isolated from a knee abscess in 1952, the prevalence of *M. abscessus* infections has been increasing worldwide. For example, in Taiwan, the proportion of AFB-positive respiratory isolates found to have NTM increased from 32.3% to 49.8% between the years 2000 and 2008, and *M. abscessus* represented 17.2% of all clinical NTM isolates [5,8]. In the US, in a study at four different healthcare centers, the prevalence of NTM isolates increased from 19.6/100,000 in the years 1994-1996, to 26.7/100,000 in the years 2004-2006 [9]. This study also found a significant increase in the prevalence of 2.6% per year among women and 2.9% per year among men [9]. Similar to the study in Taiwan, *M. abscessus* was the second most common species identified, second only to *M. avium* [9].

While the pulmonary disease may be the most significant manifestation of *M. abscesses* infection in terms of mortality, clinical presentations often include skin lesions [10]. Skin infections caused by *M. abscessus *typically manifest with red, swollen, and minimally painful subcutaneous nodules which can develop drainage tracts to the surface of the skin, and are typically associated with cosmetic procedures, trauma, and nosocomial infections [5]. Clinically, these non-specific findings can make it difficult to suspect *M. abscessus* as a causative organism, especially in the absence of identified risk factors such as trauma or cosmetic surgery. Diagnosis is often delayed until a high index of suspicion is raised by either persistent infection despite antibiotic treatment, or histologic or microbiologic findings offering a clue [11]. Histologically, the presence of vacuoles and granulomatous inflammation has been reported as a finding highly suspicious for *M. abscessus* infection and should prompt clinicians to order tissue cultures [11-14]. In this paper, we report a case of breast abscess due to *M. abscessus* in a patient with the absence of known risk factors such as trauma or cosmetic surgery, in which histological findings and cooperation between clinicians, surgical pathologists, and clinical microbiologists were key to arriving at the correct diagnosis.

## Case presentation

**Figure 1:**
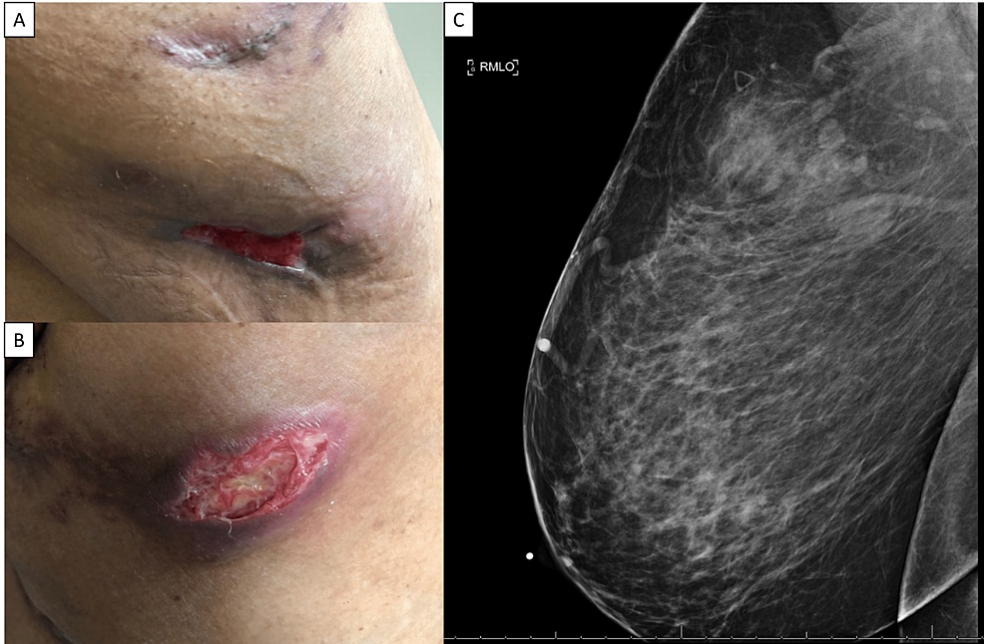
Right breast lesion. Photographs of the patient’s lesions on the right breast (A) and (B) at initial presentation. Image from the patient’s initial mammogram demonstrating a focal asymmetry in the right upper outer quadrant. This finding correlates with the palpable area of concern. Note the skin thickening associated with focal asymmetry (C).

A 58-year-old African American female presented to the Dermatology Clinic complaining of a six-month history of painful furuncles located under her right breast and on her abdomen. Her social history was significant for former tobacco use (patient quit 13 years prior to presentation), occasional social alcohol use, and no illicit drug use. The patient worked for the school district and lived in a suburban area. Her past medical history (PMH) was significant for hypothyroidism, hypertension, morbid obesity, hyperlipidemia and type 2 diabetes mellitus. She did not have a history of breast implants, reconstruction, trauma, or piercing. She reported prior treatments with oral as well as parenteral antibiotics at an outside institution, with some improvement, but the furuncles persisted. She reported pain, erythema, induration, and drainage through the skin (Figures 1A, 1B), and there was no record of the previous analysis of the drainage fluid. Given the persistence of these lesions, at the time of her presentation, there was a concern for neoplastic nodules vs. mastitis vs. abscess. She had a recent mammogram significant for fibroglandular densities and benign-appearing calcifications in the bilateral breasts, and a focal asymmetry in the right breast correlating with the area of clinical concern (Figure 1C). A follow-up breast ultrasound was performed which demonstrated an irregular hypoechoic area with posterior acoustic shadowing, likely representing a benign lesion. The patient was given a course of doxycycline 100mg by mouth twice daily for two weeks to cover for normal skin flora, but there was little improvement. The patient was then referred to surgery for incision and drainage with excisional biopsies.

**Figure 2:**
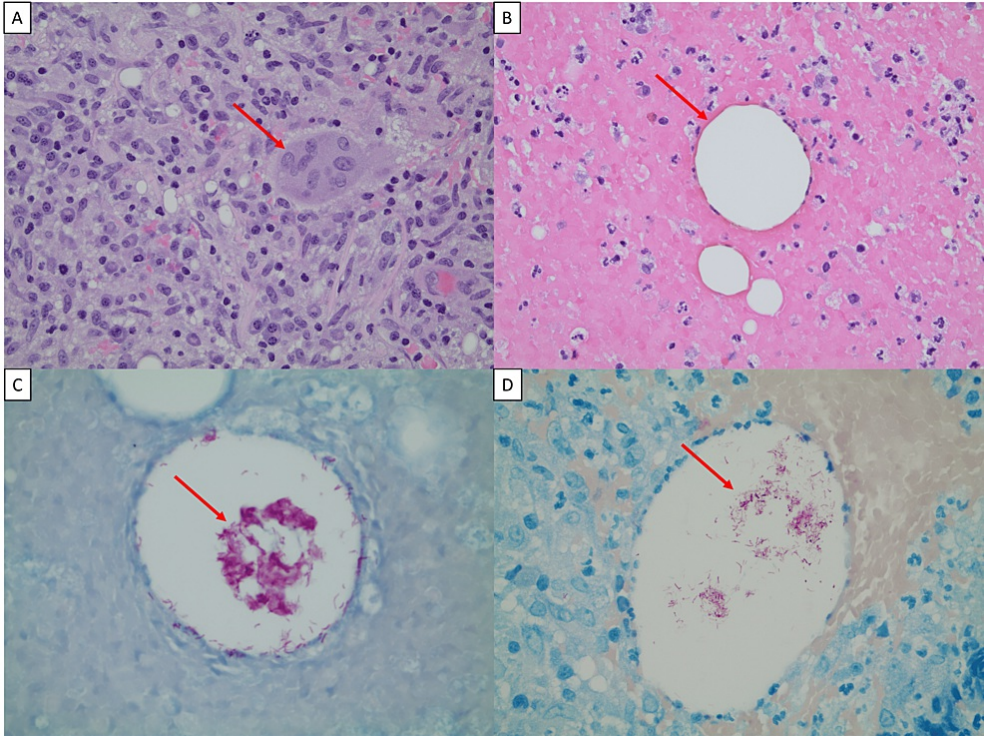
Histopathologic examination of excisional biopsy. Surgical excision of lesion at the upper outer quadrant of the right breast. (A) Granulomatous inflammation with epithelioid histiocyte, multinucleated giant cells (red arrow), lymphocytes, and plasma cells (H&E, ×400). (B) Lipid-like vacuoles (red arrow) seen in the background of acute inflammation (H&E, ×400). (C) Clumps of intravacuolar acid-fast bacilli (red arrow, AFB stain, 600). (D) FITE stain highlights acid-fast microorganisms (red arrow, FITE stain, 600).

The excisional biopsy was fixed in formalin, and paraffin embedded tissue sections were prepared per protocol. Hematoxylin and eosin (H&E)-stained sections of the tissue revealed an inflammatory infiltrate with a predominance of lymphocytes and plasma cells as well as focal neutrophils. Epithelioid histiocytes and multinucleated giant cells, indicative of granuloma formation, were also seen (Figure 2A). Interspersed within the inflammatory process were vacuolated, lipid-like spaces (Figure 2B). The lesions were noted to involve the epidermis, dermis, and subdermal tissue. No evidence of malignancy was seen on H&E-stained slides; instead, the presence of epithelioid macrophages was confirmed by CD68 stain (not shown). Due to the presence of inflammation and the absence of malignancy, Gram, Grocott’s Methenamine Silver (GMS), and Kinyoun stains were performed. Gram stain and GMS were negative, indicating that there was no significant presence of bacteria or fungi; however, the Kinyoun stain was positive for the presence of acid-fast-bacilli (AFB) organisms in the vacuolated spaces (Figure 2C). To confirm the presence of AFB, a FITE stain was performed, as this staining has been shown to be more sensitive for certain species of AFB [15], and in this case was also positive for mycobacterial organisms within the vacuoles (Figure 2D).

These findings raised our suspicions for *M. abscessus* infection [11] and were reported to the breast surgeon. The breast surgeon subsequently brought the patient back for aspiration of the skin lesion. After discussing the pathological findings with the surgeon, the specimens were sent to the microbiology laboratory for culture. Breast abscess aspirates were set up onto Middlebrook 7H10 Agar plated media (BBL, BD Diagnostic Systems, Franklin Lake, NJ), from which growth was noted by day three of incubation at 35^o^C. The organism appeared as AFB positive beaded bacilli on subsequent Kinyoun stain, and identification as *M. abscessus* was determined by Vitek MS mass spectrometry (BioMérieux, Hazelwood, MO), thus confirming the histologic diagnosis. Antimycobacterial susceptibility testing for this AFB isolate was performed through the utilization of broth microdilution minimal inhibitory concentration (MIC) methods at ARUP Laboratories (Salt Lake City, UT) and University of Texas, Tyler (Tyler, TX).

Members of *M. abscessus* are well known for broad antibiotic resistance, and appropriate treatment can be prolonged and costly. *M. abscessus* tends to be susceptible to clofazimine, amikacin, cefoxitin, and tigecycline, and resistant to imipenem, trimethoprim/sulfamethoxazole, and fluoroquinolones [16]; however, treatment regimens are dependent on susceptibility testing. Susceptibility testing was performed on our patient’s breast aspirate and showed resistance to imipenem, intermediate sensitivity to linezolid, cefoxitin, and amikacin, and sensitivity to macrolides. Our patient had complete resolution of the lesions after receiving combined therapy with oral azithromycin 600 mg by mouth (PO) daily, linezolid 600 mg PO twice daily, clofazimine 100 mg PO daily, and amikacin 1540 mg intravenously twice a week (22 mg/kg) for four months. Other adjunctive treatments were not possible due to financial constraints. Four months after completion of treatment, she had recurrence of the disease confirmed via biopsy and cultures. After negative evaluation for immunodeficiencies, malignancies, and other predisposing conditions, she is planned for total mastectomy to obtain source control and a new course of antibiotics.

## Discussion

In this paper, we present a case of NTM infection in breast tissue in the absence of known risk factors. Breast abscess, a relatively common finding in breast pathology, can be classified into lactational and non-lactational. Non-lactational abscesses are frequently identified, especially in smokers, and are typically caused by *Staphylococcus *spp or by anaerobic bacteria [17]. Prior instances of *M. abscessus* infections related to trauma and invasive cosmetic procedures, including breast prosthetic augmentation, breast reduction, liposuction, body-contour surgery, and blepharoplasty, have been described [18-22]. We are reporting a case of a subacute presentation of non-lactational breast abscesses in a woman without a history of trauma, cosmetic surgery, or breast neoplastic or non-neoplastic lesions. This unusual clinical presentation of *M. abscessus* infection can make it more difficult to diagnose and treat in a timely manner. Notably, this patient had a history of diabetes, a well-known pre-disposing factor for infection. Previous studies have moreover demonstrated an overrepresentation of diabetic patients among those with soft tissue NTM infections [23], indicating a possible predisposing factor for the observed infection.

Patients with NTM infections can have many different non-specific clinical presentations including abscesses, cysts, pustules, epidermal changes, infiltrates, fibrosis, and necrosis. Biopsy findings can be a critical component in raising suspicion about NTM infections. In immunocompetent patients, suppurative granulomas are the most reported histopathologic features observed. In immunosuppressed individuals, deeper dermal inflammation, and subcutaneous involvement as well as more suppurative infiltrate were often observed [11]. Regardless of immune status, vacuoles and granulomatous inflammation have been noted in most reported cases, although these classic histologic features are not broadly known in the breast pathology community.

The breast biopsy findings of lipid-like vacuoles and granulomatous inflammation associated with this NTM infection are of particular interest, and key to the diagnosis. The main differential for these histologic findings is cystic neutrophilic granulomatous mastitis (CNGM). CNGM is a rare breast entity, often associated with *Corynebacterium *spp. CNGM typically features a rim of neutrophils around the lipoid vacuoles, which is not commonly observed in NTM infections, and intravacuolar diphtheroid bacilli on Gram stain. The two entities can be further differentiated with AFB and FITE stains, which are negative in CNGM and positive in NTM infections [24,25]. Thus, the presence of granulomatous inflammation and positive FITE and AFB stains, in this case, is an important distinguishing characteristic, which raised our suspicion for NTM infection, and subsequently prompted clinicians to obtain cultures. The finding of vacuoles in the dermal infiltrate has been noted to be associated with *M. abscessus* in previous studies, and the organism is usually located within the vacuoles and admixed with inflammatory cells [11-14,21]. The origin of these lipid-like vacuoles remains unknown, and there are several theories about their formation. Vacuole formation in the tissue may be a result of the oxygen released from the reduction of hydrogen peroxide (H_2_­O_2_) by the catalase produced by these mycobacteria, but this remains speculative [12]. Another theory is that these vacuoles may also correspond to foci of tissue digested by polymorphonuclear leukocytes, which are then extracted during histologic processing with the persistence of the bacilli [12]. Currently, these vacuoles are widely accepted to be of lipid origin, potentially arising from fat vacuoles displaced upwards by inflammatory infiltrate [13]. It has been suggested that the lipid environment enhances the growth of the organism and provides protection from clearance by the host defenses [13].

Observation of these distinct histopathological characteristics - lipoid vacuoles, granulomatous inflammation, and intravacuolar AFB-positive bacteria - should prompt clinicians to consider NTM infection in the differential diagnosis. AFB and FITE stains were positive in our patient and highlighted the presence of organisms within lipid-like vacuoles. This classic feature is highly suggestive of NTM infection; however, due to the relatively low sensitivity of these stains, it is imperative that tissue culture and speciation are performed, and that surgical pathologists closely coordinate with the microbiology laboratory to ensure an expedient and accurate diagnosis. Clinical suspicion and identification of NTM are important as the treatment involves l, combined antibiotic therapy, along with surgical debridement for extensive infection. Moreover, NTM species, in particular *M. abscessus*, can be multidrug resistant, and developing antibiotic regimens for these organisms can be difficult and costly. Thus, close coordination between surgical pathologists, microbiologists, and clinicians is of utmost importance for timely diagnosis.

## Conclusions

Our understanding of the *Mycobacterium *genus has greatly expanded over the past decade, with increasing recognition of non-tuberculosis mycobacterium (NTM) as pathogenic agents in humans. Indeed, infections with NTM are growing worldwide, making NTM species important emerging pathogens. *M. abscessus* is known to cause both pulmonary disease and soft tissue infections, although the latter is typically associated with risk factors such as trauma or plastic surgery. Here we present a case of an NTM abscess in the breast, which is unusual for both its location and the absence of risk factors. Recognition of the underlying pathogen, in this case, depended on a careful assessment of histologic clues, namely vacuoles containing AFB-positive organisms in the setting of granulomatous infections. Of course, there are differential diagnoses that present in a similar fashion, and in this case, the appropriate diagnosis could not have been reached without close cooperation and coordination between specialties, including surgical pathology, clinical microbiology, infectious disease, and breast surgery. Thus, this case serves an important educational purpose of recognizing both clinical and histopathologic signs of *M. abscessus* infection, even in the absence of risk factors, as well as interdepartmental coordination to achieve diagnosis and treatment for a highly resistant pathogen.
